# Novel insight into the composition of human single-stranded DNA-binding protein 1 (hSSB1)-containing protein complexes

**DOI:** 10.1186/s12867-016-0077-5

**Published:** 2016-12-09

**Authors:** Nicholas W. Ashton, Dorothy Loo, Nicolas Paquet, Kenneth J. O’Byrne, Derek J. Richard

**Affiliations:** 1 School of Biomedical Research, Institute of Health and Biomedical Innovation, Queensland University of Technology, Translational Research Institute, 37 Kent Street, Woolloongabba, QLD 4102 Australia; 2 Translational Research Institute Proteomics Facility, Translational Research Institute, 37 Kent Street, Woolloongabba, QLD 4102 Australia

**Keywords:** hSSB1, mRNA metabolism, Chromatin remodelling

## Abstract

**Background:**

Single-stranded DNA-binding proteins are essential cellular components required for the protection, metabolism and processing of single-stranded DNA. Human single-stranded DNA-binding protein 1 (hSSB1) is one such protein, with described roles in genome stability maintenance and transcriptional regulation. As yet, however, the mechanisms through which hSSB1 functions and the binding partners with which it interacts remain poorly understood.

**Results:**

In this work, hSSB1 was immunoprecipitated from cell lysate samples that had been enriched for non-soluble nuclear proteins and those associating with hSSB1 identified by mass spectrometry. In doing so, 334 potential hSSB1-associating proteins were identified, with known roles in a range of distinct biological processes. Unexpectedly, whilst hSSB1 has largely been studied in a genome stability context, few other DNA repair or replication proteins were detected. By contrast, a large number of proteins were identified with roles in mRNA metabolism, reflecting a currently emerging area of hSSB1 study. In addition, numerous proteins were detected that comprise various chromatin-remodelling complexes.

**Conclusions:**

These findings provide new insight into the binding partners of hSSB1 and will likely function as a platform for future research.

**Electronic supplementary material:**

The online version of this article (doi:10.1186/s12867-016-0077-5) contains supplementary material, which is available to authorized users.

## Background

The recurrent exposure of single-stranded DNA (ssDNA) is a central aspect of cellular metabolism, permitting processes that include RNA transcription and DNA replication. Here, localised unwinding of duplex DNA is an essential initiation phase, exposing the genetic code for polymerase-mediated strand synthesis [1, 2]. ssDNA is also frequently exposed as a result of DNA damage, both as a direct result of lesion formation, as well as subsequently during repair transactions [3]. In all of these processes, ssDNA-binding proteins are required for proper manipulation of the DNA, ensuring it is maintained in a single-stranded state, while guiding the localisation of processing enzymes [4].

ssDNA-binding proteins can associate with ssDNA via a number of binding motifs. The oligonucleotide/oligosaccharide binding (OB)-fold is one such motif and is characteristic of an otherwise diverse protein family [5]. In humans, OB-fold containing proteins have important roles in processes that include replication (e.g. replication protein A [6]), DNA repair (BRCA2 [7], RMI1/2 [8], MEIOB [9], DNA ligases 1, 3 and 4 [10]), checkpoint activation (e.g. STRAP [11]), telomere maintenance (e.g. CST [12], Pot1 [13], TPP1 [14]) and protein translation (e.g. lysyl, aspartyl and asparaginyl-tRNA synthetases [15]).

Human cells also encoded two additional OB-fold containing proteins, termed human single-stranded DNA-binding proteins 1 and 2 (hSSB1 and hSSB2). Previous studies have suggested that both of these proteins function as mutually exclusive components of the sensor of single-stranded DNA (SOSS) complex in partnership with the integrator complex subunit 3 (INTS3) and hSSB-interacting protein 1 (hSSBIP1; SOSSC) [16–19]. The depletion of any of these proteins has further been demonstrated to increase the sensitivity of cells to DNA damage caused by ionising radiation exposure and treatment with the topoisomerase I inhibitor, camptothecin [17, 20, 21]. Here, hSSB1 has been reported to stimulate resection of double-strand DNA break ends by the Mre11-Nbs1-Rad50 (MRN) [22, 23] and Exo1 [24] nucleases, as well as activation of the ATM kinase [20]. Additional roles for hSSB1 have also been reported in the response to replication fork stalling [21, 25], as well as in oxidative stress repair [26, 27].

Although hSSB1 has largely been studied in relation to DNA damage repair, a recent study has indicated that hSSB1 (and hSSB2) may also function in association with the integrator complex to promote mRNA transcriptional termination at RNA polymerase II (RNA-pol II) pause sites [28]. Indeed, hSSB1 was observed to associate with RNA-pol II, as well as the transcription termination factors NELFB and SPT5.

In this study we sought to further determine the molecular function of hSSB1 by identifying additional proteins with which it may associate. These findings thereby provide new insight in the composition of hSSB1-containing protein complexes, which will likely be of assistance in directing future research.

## Results

### Immunoprecipitation of hSSB1 from samples enriched for non-soluble nuclear proteins

**Fig. 1:**
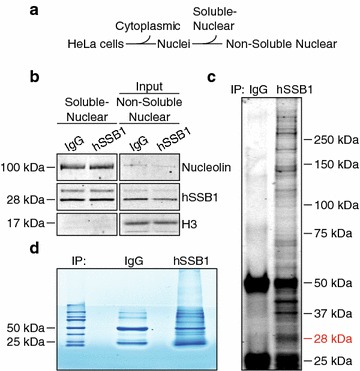
Immunoprecipitation of hSSB1 from samples enriched for non-soluble nuclear proteins. **a**, **b** HeLa cells were lysed in a buffer containing 10 mM KCl and nuclei collected by centrifugation. Soluble-nuclear proteins were then extracted from these nuclei by incubation in a buffer containing 420 mM NaCl. The remaining precipitate (containing non-soluble nuclear proteins) was digested in a buffer containing the nuclease, benzonase. The soluble and non-soluble nuclear protein fractions (10 μg) were separated by PAGE and immunoblotted with antibodies against hSSB1, nucleolin (soluble-nuclear protein marker) and H3 (chromatin-bound non-soluble nuclear protein marker). **c** 500 μg of non-soluble nuclear protein was incubated overnight at 4 °C with protein G dynabeads bound to a hSSB1 antibody, or a sheep isotype control IgG. Beads were washed five times and protein eluted by heating to 80 °C in 3× SDS loading buffer for 5 min. 10% of the eluent was separated by PAGE and stained overnight in colloidal coomassie brilliant blue G-250. The *red* 28 kDa marker indicates the migration of hSSB1. **d** The remaining eluent was resolved on a 10% acrylamide SDS-PAGE gel to a depth of 8 mM and stained overnight in colloidal coomassie brilliant blue G-250

To further elucidate the role of hSSB1 in ssDNA metabolism, we sought to identify other proteins with which hSSB1 may associate at chromatin. To achieve this, non-soluble nuclear proteins (including those bound to chromatin) were firstly enriched from HeLa cells by sub-cellular fractionation (Fig. 1a). To assess the efficacy of this technique, the soluble and non-soluble nuclear fractions were immunoblotted with antibodies against nucleolin, a protein expected to be largely soluble under these conditions [29], as well as the chromatin-associated (non-soluble) protein, histone H3 (Fig. 1b). Immunoblotting for these markers demonstrated the effective relative enrichment of non-soluble nuclear proteins in our samples.

hSSB1 was then immunoprecipitated from these samples by overnight incubation at 4 °C with protein G Dynabeads bound to hSSB1 antibodies. To determine whether proteins had been effectively co-immunoprecipitated with hSSB1, 10% of the eluted samples were separated by SDS-PAGE and stained with colloidal coomassie brilliant blue G-250 (Fig. 1c). As a number of unique bands were detected in the hSSB1 immunoprecipitated lanes when compared to the IgG lanes, this suggested the specific isolation of numerous hSSB1-associating proteins. The remaining 90% of the sample was therefore briefly separated on another SDS-PAGE gel (Fig. 1d), excised and separated into 8 equally sized fractions, digested with trypsin and analysed by liquid chromatography-coupled tandem mass spectrometry.

### Identification of hSSB1-associating proteins with a range of biological functions

The mass spectrum data collected was searched against the Swiss Prot Human database. In doing so, 334 unique proteins were identified from the hSSB1:IP sample, compared to 10 immunoprecipitated with the IgG isotype control. These 10 proteins included three keratin sub-types, two IgG molecules, two histones (one peptide each), Annexin A2, GAPDH and a member of the POTE Ankyrin domain family. The small number of proteins detected in the IgG:IP sample suggests that those identified by hSSB1 immunoprecipitation were so specifically.

**Fig. 2:**
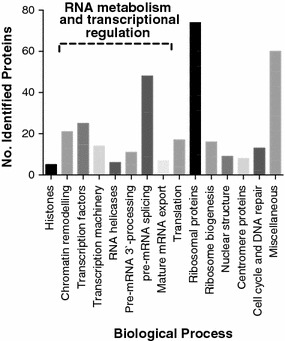
Identification of hSSB1-associating proteins with a range of biological functions. The gel-embedded, coomassie blue-stained proteins in Fig. 1d were divided into eight 1 mM gel slices, digested with trypsin and extracted. Peptides were separated and detected using an Agilent HPLC CHIP QTOF 6530 system. The mass spectrum data was extracted and searched against the Swiss Prot Human database. Proteins for which corresponding peptides were identified, as well as the number of unique detected peptides, are given in Additional file 1. Proteins were manually sorted based on their predominant known biological process as given by UniProt (http://www.uniprot.org). Proteins identified from the hSSB1:IP sample are summarised as the number of unique proteins identified for each biological process

Interestingly, despite the major known role of hSSB1 in the maintenance of genome stability, only a relatively small number of the hSSB1-associating proteins identified are known to function in either DNA repair or replication (Fig. 2; Table 1; Additional file 1). These include the minichromosome maintenance complex subunits 6 and 7 (MCM6 and MCM7), both of which form part of the helicase complex required for unwinding duplex DNA during replication [30]. In addition, numerous peptides were identified belonging to DNA topoisomerase II alpha, an enzyme that alters the superhelical state of DNA during both replication and mRNA transcription [31]. A number of peptides were also detected corresponding to CUL4A and DDB1, which function together with other DDB1-Cul4-X-box (DCX) E3 ubiquitin ligase components to initiate DNA repair signalling following UV-induced DNA damage [32].

**Table 1:** Summarising select proteins for which corresponding peptides were identified with roles in DNA repair and replication

Protein	# Unique peptides	% Coverage
CUL4A	6	10.2
DDB1	4	7.9
TOP2A	14	10.1
MCM6	1	1.8
MCM7	1	1.2

The largest group of proteins identified in our dataset contained 77 ribosomal proteins, belonging either to the 40S or 60S subunit. In addition, 23 proteins involved in ribosome biosynthesis were also detected (Fig. 2). These proteins were likely identified due to the isolation of nucleoli following the nuclear lysis step of the subcellular fractionation, proteins of which may have been liberated during the subsequent incubation. RNA metabolism proteins were also highly represented, including a number of components of the cleavage and polyadenylation specificity factor complex (CPSF; CPSF components 2, 5, 7 and 7), as well as numerous other ancillary proteins involved in polyadenylation and 3′-end cleavage of mammalian pre-mRNAs. A number of proteins known to promote pre-mRNA splicing were also detected and were represented by subunits and interacting partners of the U4/U6-U5 tri-snRNP complex, as well as other spliceosome factors. Numerous ATP-dependent RNA helicases were also detected (Table 2; DDX5, DHX15), many of which are of unknown physiological function, although include subunits of the exon junction complex, which marks the position of exon–exon junctions in mature mRNA and promotes mRNA export and translation [33]. Components of heterogeneous nuclear ribonucleoprotein (hnRNP) complexes were also detected which provide similar pre-mRNA processing functions to those mentioned above [34].

**Table 2:** Summarising select proteins for which corresponding peptides were identified with roles in mRNA processing

Protein	# Unique peptides	% Coverage
U5-116 kDa	19	33.0
SF3B1	16	18.9
DDX5	14	27.0
DHX15	12	22.7
HNRNPUL2	13	22.2
HNRNPC	12	38.2
HNRNPM	10	22.6
HNRNPK	8	30.6

Consistent with prior findings regarding a role for hSSB1 in Integrator-mediated transcriptional termination [28], components of both the Integrator and RNA polymerase II complexes were also identified (Table 3), supporting such a function for hSSB1, as well as the validity of our dataset.

**Table 3:** Summarising select proteins for which corresponding peptides were identified which have previously been described as associating with hSSB1

Protein	# Unique peptides	% Coverage
INTS1	1	0.5
INTS3	5	7.9
INTS5	1	1.3
RPB1	2	1.8
RPB2	5	6.1
RPB3	3	14.1
RPB5	1	8.0
RPB9	1	18.4

### hSSB1 associates with proteins comprising numerous chromatin-remodelling complexes

Numerous proteins involved in chromatin remodelling and modification were also detected in the mass spectrometry dataset and represent a number of known protein complexes (Table 4).

**Table 4:** From the proteins grouped in the ‘chromatin remodelling’ biological process in Fig. 2, components of numerous chromatin-remodelling complexes were identified

NuRD	WICH-ISWI	SWI/SNF	Tip60 (NuA4)	SIN3
*MTA2*	*BAZ1B*	*β-actin*	*ACTL6A*	*HDAC1*
*RBBP4*	*SMARCA5*	*ACTL6A*	*RUVBL1*	*HDAC2*
*RBBP7*		*BRG1*	RUVBL2	*RBBP4*
*HDAC1*		*PBRM1*	HTATIP	*RBBP7*
*HDAC2*		SMARCB1	TRRAP	SAP18
CHD3		*SMARCC1*		SIN3A
*CHD4*		SMARCC2		SAP30
MBD2		SMARCD1		
*MBD3*		*SMARCD2*		
		*SMARCE1*		
		DPF2		
		ARID5A/B/C		
		BCL7		
		BRD7		
		BCL11B		

Protein components of each identified complex are listed in columns and those identified in the hSSB1:IP dataset shown in italics font

These include the WICH complex components BAZ1B and SMARCA5, as well as the NuRD complex components MTA2 and RBBP4 (Table 5), each of which were amongst our highest ‘hits’.

**Table 5:** Summarising the number of unique peptides identified, as well as the percentage protein coverage, for selected chromatin remodelling complex proteins identified by mass spectrometry in the hSSB1:IP sample

Protein	# Unique peptides	% Coverage
BAZ1B/WSTF	17	15.5
SMARCA5/SNF2H	16	15.4
MTA2	16	29
RBBP4/RBAP48	15	58.8

**Fig. 3:**
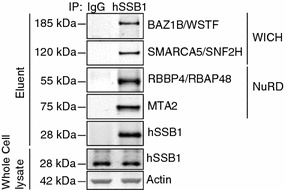
hSSB1 associates with proteins comprising numerous chromatin-remodelling complexes. HeLa whole cell lysates were prepared and 500 μg incubated for 2 h at 4 °C with protein G dynabeads bound to a hSSB1 antibody, or a sheep isotype control IgG. Beads were washed five times and protein eluted by heating to 80 °C in 3× SDS loading buffer for 5 min. Eluent was immunoblotted with antibodies against BAZ1B, SMARCA5, MTA2, RBBP4 and hSSB1 as indicated. 15 μg of whole cell lysate (input) was immunoblotted with antibodies against hSSB1 and actin (loading control)

To further validate the association of hSSB1 with WICH and NuRD complex proteins, hSSB1 was immunoprecipitated from HeLa whole cell lysates and co-eluting proteins immunoblotted with antibodies against BAZ1B, SMARCA5, MTA2 and RBBP4 (Fig. 3). Consistent with the mass spectrometry data, each of these proteins was specifically immunoprecipitated with hSSB1. These data thereby support that hSSB1 associates with these chromatin remodelling complexes in cells.

## Discussion

The data presented in this study suggests that while hSSB1 has predominantly been considered in a DNA repair context, it is likely to have roles in many other cellular processes. In particular, the identification of numerous proteins with roles in mRNA metabolism, transcriptional transactions and ribosomal processes, may suggest these as important areas of hSSB1 function. These findings are supportive of recent work describing a role for hSSB1 in transcriptional termination, as well as the likely association of hSSB1 with proteins of the integrator and RNA polymerase II complex [28]. Consistent with the observation that hSSB1 may be required for replication-dependent histone mRNA processing [28], we also identified proteins of the cleavage and polyadenylation specificity factor complex (CPSF), components of which have been attributed to this and other processes [35]. Further, the identification of hSSB1-associating proteins with known roles during earlier and later stages of gene expression (e.g. transcriptional activation and repression, mRNA splicing, processing and transport) may suggest additional processes in which hSSB1 could also function.

The association of hSSB1 with a large number of ribosomal proteins and translation elongation factors (especially eukaryotic translation initiation factor 3 subunits) may also suggest a novel role in protein translation. In this case, it is possible that membrane-bound ribosomes may have been co-isolated with other non-soluble proteins during fractionation of HeLa cell lysates prior to mass spectrometry analysis. As numerous nucleolar and ribosome biogenesis proteins were also identified, an alternative explanation may include the detection of hSSB1-associating ribosomal proteins due to the co-isolation of nucleoli. As yet, however, the localisation of hSSB1 to either of these structures remains untested and will require verification via alternative approaches. In addition, due to the high abundance of ribosomal components in the cell, these proteins are frequent contaminants of mass spectrometry datasets that have been generated from affinity isolated samples [36]. We therefore cannot exclude the possibility that although these proteins were not identified in our IgG sample, their detection may be due to non-specific electrostatic interaction with hSSB1 or other proteins. Indeed, the inability to discriminate genuine interacting proteins from those that have non-specifically associated with hSSB1 during immunoprecipitation represents a limitation of these assays. In future work, the validation of hSSB1 associations may therefore benefit from use of additional technical approaches, such as proximity ligation assays, which do not require prior cell lysis. In addition, it will be important to establish the physiological significance of hSSB1 in those pathways suggested in Fig. 2, which may reinforce the validity of our data by indirectly reflecting the associations described in this manuscript. For instance, it will be important to assess the physiological role of hSSB1 in chromatin remodelling. This may be particularly rewarding given that chromatin-remodelling complexes have been suggested to function in replication fork integrity and DNA repair [37–39], as well as have an essential role in mRNA transcription [40]. A functional, physiological role for hSSB1 with these proteins may therefore suggest a novel means through which hSSB1 influences gene expression and genome stability in cells.

## Conclusions

The findings described here provide insight into the composition of hSSB1-containing protein complexes. These associations suggest functional partners through which hSSB1 may promote genome stability maintenance and transcriptional regulation, as well as indicate other biological processes in which hSSB1 may participate. The continued characterisation of these findings will likely yield a greater understanding of hSSB1 molecular function.

## Methods

### Cell culture

HeLa cells were obtained from the American Type Culture Collection (ATCC) and maintained in Roswell Park Memorial Institute Medium (RPMI, Sigma-Aldrich) supplemented with 10% foetal bovine serum (Sigma-Aldrich) and cultured at 37 °C in a humidified incubator with 5% CO_2_.

### Subcellular fractionation

Subcellular fractionation was performed using an adaptation of a previously described methodology [41]. Here, cells were resuspended in Buffer A (20 mM HEPES pH 7.9, 10 mM MgCl_2_, 10 nM KCl, 0.05 mM DTT, 0.05% Triton X-100, 1× protease inhibitors 1× phosphatase inhibitors) vortexed at maximum speed for 5 s, then incubated at 4 °C with agitation for 10 min before passing through a 26 gauge needle 6 times. Solutions were then centrifuged at 500×*g* for 10 min at 4 °C and the supernatant (cytoplasmic fraction) removed. Nuclei were washed once in Buffer A before resuspension in Buffer C (20 mM HEPES pH 7.9, 10 mM MgCl_2_, 0.05 mM EDTA, 420 mM NaCl, 25% glycerol, 0.05% Triton X-100, 1× protease inhibitors 1× phosphatase inhibitors). Solutions were vortexed at maximum speed for 15 s, then incubated at 4 °C with agitation for 30 min, before centrifugation at 2000×*g* for 10 min at 4 °C. Supernatant (soluble nuclear fraction) was collected and the pellet washed once with Buffer C. The pellet was resuspended in Buffer C containing 4000 units of micrococcal nuclease, vortexed for 15 s and incubated for 30 min at room temperature with agitation. Solutions were centrifuged at 21,000×*g* for 10 min and the supernatant (non-soluble nuclear fraction) collected.

### Antibodies and immunoblotting

Electrophoresis (4–12% Bis–Tris Plus Bolt precast gels; ThermoFisher) was employed for the separation of whole cell lysate, subcellular fraction or eluent samples. Proteins were then transferred to nitrocellulose and immunoblotted. Commercial antibodies against nucleolin (clone D4C7O, cat# 14574) and H3 (clone D1H2, cat# 4499) were purchased from Cell Signaling Technology. The BAZ1B/WSTF (clone EP1704Y, cat# ab51256), SMARCA5/SNF2H (cat# ab3749), RBBP4/RBAP48 (cat# ab1765) and MTA2 (cat# ab8106) antibodies were purchased from Abcam. The actin antibody (clone C4, cat# 612656) was purchased from BD Biosciences, whilst the hSSB1 antibody was purified from sheep anti-serum as has been described previously [20]. IRDye 680RD or 800CW-conjugated donkey anti-mouse, rabbit or goat fluorescent secondary antibodies (Li-Cor) were used for the visualisation of primary antibodies with the Odyssey Imaging System (Li-Cor).

### Immunoprecipitation

For immunoprecipitation from non-soluble nuclear protein fractions, samples were firstly diluted with equal volumes of Buffer A. For immunoprecipitation from whole cell lysates, cells were resuspended in immunoprecipitation buffer (20 mM HEPES pH 7.5, 150 mM KCl, 5% glycerol, 10 mM MgCl_2_, 0.5% Triton X-100) supplemented with 1× phosphatase inhibitor cocktail and 1x protease inhibitor cocktail and then lysed by sonication (3 × 3 s bursts, 10% output; Vibra-Cell, 3 mm probe; Sonics and Materials). Prior to hSSB1 immunoprecipitation, anti-hSSB1 or sheep IgG isotype control (Sigma-Aldrich) antibodies were coupled with magnetic protein G Dynabeads (ThermoFisher). Beads were then incubated with protein samples either for 2 h or overnight at 4 °C, washed 5 times and proteins eluted by incubation in 3× SDS loading dye at 80 °C for 5 min.

### Mass spectrometry and data analysis

Eluent samples were separated on a 10% acrylamide Mini-PROTEAN TGX precast SDS-PAGE Gel (Bio-Rad, Gladesville, NSW) to a depth of 8 mM. The gel was stained with colloidal coomassie brilliant blue G-250 and the sample divided into eight 1 mM gel bands. In-gel digestion was performed using an Agilent Bravo automated liquid handling platform (Agilent Technologies, Mulgrave, Victoria) to achieve the following: 90 min incubation in de-stain buffer (50% v/v acetonitrile, 25 mM NH_4_HCO_3_), dehydration for 10 min using a SpeedVac, 30 min incubation in reducing buffer (20 mM Dithiothreitol, 50 mM NH_4_HCO_3_) at 37 °C, 20 min incubation in alkylation buffer (50 mM iodoacetamide) in the dark, dehydration for 10 min using a SpeedVac, overnight trypsin digestion (0.4 μg trypsin, 10% acetonitrile, 25 mM NH_4_HCO_3_), trypsin inactivation with 5% formic acid and peptide elution in extraction buffer (1% formic acid, 60% acetonitrile). Peptides were dried using a SpeedVac, resuspended in 5% formic acid and analysed using an Agilent HPLC CHIP QTOF 6530 system (Agilent Technologies). Peptides were loaded onto an Agilent (Agilent Technologies) G4240-62010 large capacity chip in a solution containing 0.1% formic acid and 90% acetonitrile with a flow rate of 2.5 μl min^−1^. Peptides were separated with a gradient of 0.1% formic acid, 3% acetonitrile to 0.1% formic acid, 36% acetonitrile. The MS1 analyzer acquired ions from 100 to 1700 m/z at a rate of 10 spectra/s. The MS2 analyzer acquired ions from 50 to 1700 m/z at a rate of 3 spectra/s. A maximum of 10 precursors were selected per cycle and ions were excluded after 1 spectra and released after 15 s. Data obtained in this way was processed using Spectrum Mill (Agilent technologies, B.04.00.127) and extracted data searched against the Swiss-Prot Human (version 11/2014) database. Search parameters included trypsin digestion, carbamidomethyl of cysteine residues as fixed modification and methionine oxidation as variable modification. Protein identification was summarised from peptides with less than 0.5% false discovery rate.

## Additional file

**Additional file 1: Table S1.** hSSB1-associating proteins for which corresponding peptides were identified by mass-spectrometry, as well as the number of unique peptides detected. Proteins are grouped based on their predominant known biological process as given by UniProt (http://www.uniprot.org).

## Abbreviations

CPSF: cleavage and polyadenylation specificity factor
DCX: DBB1-Cul4-X-box
hnRNP: heterogeneous nuclear riboprotein
hSSB1: human single-stranded DNA-binding protein 1
hSSBIP1: hSSB-interacting protein 1
INTS3: integrator complex subunit 3
MCM: minichromosome maintenance complex
MRN: Mre11-Rad50-Nbs1
OB: oligonucleotide/oligosaccharide binding
RPA: replication protein A
SOSS: sensor of single-stranded DNA
ssDNA: single-stranded DNA
