# Supplementary material for: Novel insight into the composition of human single-stranded DNA-binding protein 1 (hSSB1)-containing protein complexes
Source: BMC Mol Biol. 2016 Dec 9;17:24. doi: 10.1186/s12867-016-0077-5 (PMC5148904; doi:10.1186/s12867-016-0077-5)
Supplement: Supplementary file 1 — Additional file 1: Table S1. hSSB1-associating proteins for which corresponding peptides were identified by mass-spectrometry, as well as the number of unique peptides detected. Proteins are grouped based on their predominant known biological process as given by UniProt (http://www.uniprot.org). [file 12867_2016_77_MOESM1_ESM.docx]

**Table S1:** hSSB1-associating proteins for which corresponding peptides were identified by mass-spectrometry, as well as the number of unique peptides detected. Proteins are grouped based on their predominant known biological process as given by UniProt (www.uniprot.org).

| Protein | # Unique peptides | Protein | # Unique peptides |
| --- | --- | --- | --- |
| **Histones** | | | |
| Core histone macro-H2A.1 | 14 | Histone H3.2 | 5 |
| Histone H2A type 2-A | 6 | Histone H4 | 9 |
| Histone H2B type 1-B | 6 |  |  |
| **Chromatin Remodelling/Modifications** | | | |
| Actin-like protein 6A | 2 | Protein polybromo-1 | 2 |
| Actin, beta | 20 | Protein Wiz | 1 |
| Chromodomain-helicase-DNA-binding protein 4 | 3 | Remodeling and spacing factor 1 | 1 |
| Histone acetyltransferase KAT7 | 1 | RuvB-like 1 | 5 |
| Histone deacetylase 1 / Histone deacetylase 2 | 9 | SWI/SNF complex subunit SMARCC1 | 3 |
| Histone-binding protein RBBP4 / Histone-binding protein RBBP7 | 14 | SWI/SNF-related matrix-associated actin-dependent regulator of chromatin subfamily A member 5 / Probable global transcription activator SNF2L1 | 16 |
| Histone-lysine N-methyltransferase EHMT1 | 2 | SWI/SNF-related matrix-associated actin-dependent regulator of chromatin subfamily D member 2 | 1 |
| Histone-lysine N-methyltransferase EHMT2 | 1 | SWI/SNF-related matrix-associated actin-dependent regulator of chromatin subfamily E member 1 | 2 |
| Metastasis-associated protein MTA2 / Metastasis-associated protein MTA1/ Metastasis-associated protein MTA3 | 16 | Transcription activator BRG1 | 3 |
| Methyl-CpG-binding domain protein 3 | 1 | Tyrosine-protein kinase BAZ1B | 17 |
| Polycomb protein SUZ12 | 1 |  |  |
| **Transcription Factors, Activators and Repressors** | | | |
| Activity-dependent neuroprotector homeobox protein | 10 | Polymerase I and transcript release factor | 2 |
| Alpha-actinin-4 | 1 | Proline-, glutamic acid- and leucine-rich protein 1 | 15 |
| ATPase family AAA domain-containing protein 2 | 6 | Protein C3orf33 | 1 |
| Bcl-2-associated transcription factor 1 | 4 | RNA-binding protein 14 | 2 |
| Catenin delta-1 | 1 | SAFB-like transcription modulator | 2 |
| Cell division cycle 5-like protein | 2 | Scaffold attachment factor B1 / Scaffold attachment factor B2 | 12 |
| Chromobox protein homolog 3 / Chromobox protein homolog 5 / Chromobox protein homolog 1 | 8 | Serrate RNA effector molecule homolog | 2 |
| Chromodomain Y-like protein | 1 | Testis-expressed sequence 10 protein | 10 |
| High mobility group protein 20A | 3 | Transcription factor ETV6 | 1 |
| M-phase phosphoprotein 8 | 2 | Transcription intermediary factor 1-alpha | 10 |
| Periphilin-1 | 1 | Transcriptional repressor p66-beta / Transcriptional repressor p66-alpha | 7 |
| PHD finger protein 14 | 1 |  |  |
| **Transcription** | | | |
| DBIRD complex subunit ZNF326 | 2 | Integrator complex subunit 3 | 5 |
| DNA-directed RNA polymerase II subunit RPB1 | 2 | Integrator complex subunit 5 | 1 |
| DNA-directed RNA polymerase II subunit RPB2 | 5 | Regulation of nuclear pre-mRNA domain-containing protein 2 | 4 |
| DNA-directed RNA polymerase II subunit RPB3 | 3 | Transcription elongation factor SPT6 | 5 |
| DNA-directed RNA polymerase II subunit RPB9 | 1 | Zinc finger CCCH domain-containing protein 14 | 2 |
| DNA-directed RNA polymerases I, II, and III subunit RPABC1 | 1 | Zinc finger CCCH domain-containing protein 18 | 1 |
| Integrator complex subunit 1 | 1 |  |  |
| **RNA Helicases** | | | |
| ATP-dependent RNA helicase DDX18 | 2 | Probable ATP-dependent RNA helicase DDX5 / Probable ATP-dependent RNA helicase DDX17 | 14 |
| ATP-dependent RNA helicase DDX50 | 1 | Eukaryotic initiation factor 4A-III | 8 |
| Putative pre-mRNA-splicing factor ATP-dependent RNA helicase DHX15 | 12 | U5 small nuclear ribonucleoprotein 200 kDa helicase | 5 |
| **Pre-mRNA 3`-Processing** | | | |
| Cleavage and polyadenylation specificity factor subunit 2 | 2 | WD repeat-containing protein 18 | 3 |
| Cleavage and polyadenylation specificity factor subunit 5 | 2 | WD repeat-containing protein 43 | 1 |
| Cleavage and polyadenylation specificity factor subunit 6 | 2 | Pre-mRNA 3'-end-processing factor FIP1 | 2 |
| Cleavage and polyadenylation specificity factor subunit 7 | 1 | Protein virilizer homolog | 4 |
| Cleavage stimulation factor subunit 2 | 1 | Symplekin | 6 |
| ELAV-like protein 1 | 1 |  |  |
| **Pre-mRNA Splicing** | | | |
| 116 kDa U5 small nuclear ribonucleoprotein component | 15 | RNA-binding protein Raly | 6 |
| Apoptotic chromatin condensation inducer in the nucleus | 14 | Serine/arginine-rich splicing factor 1 | 10 |
| Double-stranded RNA-specific adenosine deaminase | 1 | Serine/arginine-rich splicing factor 10 | 4 |
| Heat shock cognate 71 kDa protein | 12 | Serine/arginine-rich splicing factor 7 | 5 |
| Heterogeneous nuclear ribonucleoprotein A3 | 5 | Serine/arginine-rich splicing factor 9 | 7 |
| Heterogeneous nuclear ribonucleoprotein H | 7 | Small nuclear ribonucleoprotein Sm D1 | 2 |
| Heterogeneous nuclear ribonucleoprotein H3 | 4 | Small nuclear ribonucleoprotein Sm D2 | 2 |
| Heterogeneous nuclear ribonucleoprotein K | 8 | Small nuclear ribonucleoprotein-associated protein N | 2 |
| Heterogeneous nuclear ribonucleoprotein L | 2 | SNW domain-containing protein 1 | 4 |
| Heterogeneous nuclear ribonucleoprotein M | 10 | Splicing factor 3A subunit 1 | 2 |
| Heterogeneous nuclear ribonucleoprotein U-like protein 1 | 1 | Splicing factor 3A subunit 3 | 2 |
| Heterogeneous nuclear ribonucleoprotein U-like protein 2 | 13 | Splicing factor 3B subunit 1 | 16 |
| Heterogeneous nuclear ribonucleoproteins C1/C2 | 12 | Splicing factor 3B subunit 2 | 7 |
| Intron-binding protein aquarius | 1 | Splicing factor 3B subunit 3 | 1 |
| KH domain-containing, RNA-binding, signal transduction-associated protein 1 | 1 | Splicing factor 3B subunit 4 | 1 |
| Matrin-3 | 14 | Splicing factor U2AF 65 kDa subunit | 6 |
| Non-POU domain-containing octamer-binding protein | 1 | Splicing factor, proline- and glutamine-rich | 2 |
| Nuclease-sensitive element-binding protein 1 | 1 | Transformer-2 protein homolog alpha | 2 |
| Pre-mRNA-processing factor 6 | 1 | Transformer-2 protein homolog beta | 2 |
| Pre-mRNA-processing-splicing factor 8 | 1 | U2 snRNP-associated SURP motif-containing protein | 2 |
| Pre-mRNA-splicing regulator WTAP | 1 | U4/U6 small nuclear ribonucleoprotein Prp31 | 2 |
| Protein mago nashi homolog 2 | 4 | U4/U6.U5 tri-snRNP-associated protein 1 | 1 |
| RNA-binding motif protein, X chromosome | 9 | U5 small nuclear ribonucleoprotein 40 kDa protein | 4 |
| RNA-binding protein 12B | 2 |  |  |
| **Mature mRNA Export** | | | |
| Ataxin-2-like protein | 3 | RNA-binding protein 8A | 2 |
| Caprin-1 | 1 | THO complex subunit 5 homolog | 1 |
| Nuclear cap-binding protein subunit 1 | 4 | THO complex subunit 6 homolog | 1 |
| Plasminogen activator inhibitor 1 RNA-binding protein | 1 |  |  |
| **Translation** | | | |
| Elongation factor 1-alpha 1 | 1 | Eukaryotic translation initiation factor 3 subunit L | 6 |
| Elongation factor 1-gamma | 1 | Eukaryotic translation initiation factor 3 subunit M | 3 |
| Eukaryotic translation initiation factor 3 subunit A | 11 | Eukaryotic translation initiation factor 4B | 1 |
| Eukaryotic translation initiation factor 3 subunit B | 1 | Fragile X mental retardation protein 1 | 2 |
| Eukaryotic translation initiation factor 3 subunit C-like protein | 1 | Fragile X mental retardation syndrome-related protein 1 | 2 |
| Eukaryotic translation initiation factor 3 subunit E | 2 | La-related protein 1 | 4 |
| Eukaryotic translation initiation factor 3 subunit F | 3 | Polyadenylate-binding protein 1 | 5 |
| Eukaryotic translation initiation factor 3 subunit G | 3 | Polypyrimidine tract-binding protein 1 | 1 |
| Eukaryotic translation initiation factor 3 subunit I | 1 |  |  |
| **Ribosomal Proteins** | | | |
| 28S ribosomal protein S27, mitochondrial | 1 | 60S ribosomal protein L12 | 5 |
| 39S ribosomal protein L38, mitochondrial | 1 | 60S ribosomal protein L13 | 6 |
| 40S ribosomal protein S10 | 2 | 60S ribosomal protein L13a | 5 |
| 40S ribosomal protein S11 | 2 | 60S ribosomal protein L14 | 3 |
| 40S ribosomal protein S12 | 5 | 60S ribosomal protein L15 | 5 |
| 40S ribosomal protein S13 | 4 | 60S ribosomal protein L17 | 5 |
| 40S ribosomal protein S14 | 4 | 60S ribosomal protein L18 | 5 |
| 40S ribosomal protein S15 | 1 | 60S ribosomal protein L18a | 5 |
| 40S ribosomal protein S15a | 2 | 60S ribosomal protein L19 | 3 |
| 40S ribosomal protein S16 | 5 | 60S ribosomal protein L21 | 1 |
| 40S ribosomal protein S17 | 6 | 60S ribosomal protein L22 | 3 |
| 40S ribosomal protein S18 | 8 | 60S ribosomal protein L23 | 1 |
| 40S ribosomal protein S19 | 4 | 60S ribosomal protein L23a | 3 |
| 40S ribosomal protein S2 | 10 | 60S ribosomal protein L24 | 3 |
| 40S ribosomal protein S20 | 1 | 60S ribosomal protein L26 | 2 |
| 40S ribosomal protein S21 | 1 | 60S ribosomal protein L27 | 3 |
| 40S ribosomal protein S23 | 1 | 60S ribosomal protein L27a | 4 |
| 40S ribosomal protein S24 | 3 | 60S ribosomal protein L28 | 2 |
| 40S ribosomal protein S25 | 3 | 60S ribosomal protein L29 | 1 |
| 40S ribosomal protein S26 | 3 | 60S ribosomal protein L3 | 11 |
| 40S ribosomal protein S27-like | 1 | 60S ribosomal protein L30 | 3 |
| 40S ribosomal protein S28 | 2 | 60S ribosomal protein L31 | 1 |
| 40S ribosomal protein S3 | 13 | 60S ribosomal protein L32 | 1 |
| 40S ribosomal protein S3a | 6 | 60S ribosomal protein L34 | 2 |
| 40S ribosomal protein S4, X isoform | 10 | 60S ribosomal protein L35 | 1 |
| 40S ribosomal protein S5 | 2 | 60S ribosomal protein L35a | 2 |
| 40S ribosomal protein S6 | 5 | 60S ribosomal protein L36 | 2 |
| 40S ribosomal protein S7 | 7 | 60S ribosomal protein L36a-like | 1 |
| 40S ribosomal protein S8 | 7 | 60S ribosomal protein L37a | 1 |
| 40S ribosomal protein S9 | 7 | 60S ribosomal protein L4 | 14 |
| 40S ribosomal protein SA | 13 | 60S ribosomal protein L5 | 7 |
| 60S acidic ribosomal protein P0 | 13 | 60S ribosomal protein L6 | 9 |
| 60S acidic ribosomal protein P1 | 2 | 60S ribosomal protein L7 | 12 |
| 60S acidic ribosomal protein P2 | 4 | 60S ribosomal protein L7a | 7 |
| 60S ribosomal protein L10 | 6 | 60S ribosomal protein L8 | 6 |
| 60S ribosomal protein L10a | 5 | 60S ribosomal protein L9 | 3 |
| 60S ribosomal protein L11 | 2 |  |  |
| **Ribosome Biogenesis** | | | |
| Bystin | 1 | Probable rRNA-processing protein EBP2 | 7 |
| Nucleolar complex protein 2 homolog | 2 | Ribosomal biogenesis protein LAS1L | 2 |
| Nucleolar GTP-binding protein 1 | 4 | Ribosomal L1 domain-containing protein 1 | 6 |
| Nucleolar GTP-binding protein 2 | 2 | Ribosome biogenesis protein BRX1 homolog | 9 |
| Nucleolar protein 58 | 1 | Ribosome biogenesis regulatory protein homolog | 1 |
| Nucleolar protein 6 | 2 | rRNA 2'-O-methyltransferase fibrillarin | 1 |
| Nucleophosmin | 4 | Sentrin-specific protease 3 | 1 |
| Polynucleotide 5'-hydroxyl-kinase NOL9 | 3 | Superkiller viralicidic activity 2-like 2 | 3 |
| **Cell Cycle and DNA Repair** | | | |
| Cullin-4A | 6 | Serine/threonine-protein phosphatase PP1-beta catalytic subunit | 2 |
| DNA damage-binding protein 1 | 4 | Cyclin-dependent kinase inhibitor 2A, isoform 4 | 2 |
| DNA replication licensing factor MCM6 | 1 | Tyrosine-protein kinase Yes | 1 |
| DNA replication licensing factor MCM7 | 1 | Enhancer of rudimentary homolog | 2 |
| DNA topoisomerase 2-alpha / DNA topoisomerase 2-beta | 14 | NEDD8 | 2 |
| E3 ubiquitin-protein ligase RING1 | 1 | Transitional endoplasmic reticulum ATPase | 3 |
| Serine/threonine-protein kinase mTOR | 1 |  |  |
| **Centromere Proteins and Regulation** | | | |
| Aurora kinase B | 2 | Kinesin-like protein KIF23 | 1 |
| Borealin | 2 | MAU2 chromatid cohesion factor homolog | 3 |
| Centromere protein V | 1 | Mitotic interactor and substrate of PLK1 | 1 |
| Emerin | 2 | Structural maintenance of chromosomes protein 1A | 1 |
| **Nuclear Structure and Scaffolding Proteins** | | | |
| A-kinase anchor protein 8 | 1 | LIM domain and actin-binding protein 1 | 1 |
| Barrier-to-autointegration factor | 2 | Nuclear mitotic apparatus protein 1 | 1 |
| Flotillin-1 | 4 | Prelamin-A/C | 8 |
| Flotillin-2 | 2 | Vimentin | 1 |
| Lamin-B1 | 1 |  |  |
| **Miscellaneous** | | | |
| 4F2 cell-surface antigen heavy chain | 3 | Junction plakoglobin | 1 |
| 60 kDa heat shock protein, mitochondrial | 1 | Kelch-like protein 35 | 1 |
| Alkaline phosphatase, tissue-nonspecific isozyme | 2 | Keratin, type I cytoskeletal 10 | 10 |
| Annexin A2 | 6 | Keratin, type I cytoskeletal 9 | 5 |
| ATP synthase subunit alpha, mitochondrial | 2 | Keratin, type II cytoskeletal 1 | 13 |
| ATP synthase subunit beta, mitochondrial | 1 | Keratin, type II cytoskeletal 7 | 1 |
| ATP-binding cassette sub-family E member 1 | 1 | Mitochondrial carrier homolog 2 | 1 |
| BAG family molecular chaperone regulator 2 | 1 | Myb-binding protein 1A | 3 |
| Brain acid soluble protein 1 | 3 | Myosin-9 | 4 |
| Caveolin-1 | 2 | Nebulin | 1 |
| CD109 antigen | 4 | Peroxiredoxin-1 | 1 |
| CD44 antigen | 2 | Peroxiredoxin-2 | 1 |
| CD59 glycoprotein | 2 | PH-interacting protein | 1 |
| Coiled-coil domain-containing protein 25 | 1 | Protein FAM208A | 5 |
| Complement component 1 Q subcomponent-binding protein, mitochondrial | 2 | Protein kinase C and casein kinase substrate in neurons protein 3 | 1 |
| Complement decay-accelerating factor | 3 | Protein Red | 1 |
| Desmoglein-2 | 3 | PWWP domain-containing protein MUM1L1 | 1 |
| Dihydrolipoyllysine-residue succinyltransferase component of 2-oxoglutarate dehydrogenase complex, mitochondrial | 2 | Ras GTPase-activating protein-binding protein 2 | 2 |
| Dolichyl-diphosphooligosaccharide--protein glycosyltransferase 48 kDa subunit | 2 | Solute carrier family 12 member 2 | 1 |
| Drebrin | 2 | Stomatin-like protein 2, mitochondrial | 2 |
| Erlin-1 | 1 | SUN domain-containing protein 2 | 1 |
| Erythrocyte band 7 integral membrane protein | 5 | Thy-1 membrane glycoprotein | 1 |
| Glioma tumor suppressor candidate region gene 2 protein | 1 | Titin | 1 |
| Glyceraldehyde-3-phosphate dehydrogenase | 1 | tRNA-splicing ligase RtcB homolog | 1 |
| Glypican-1 | 1 | Tubulin alpha-1B chain | 1 |
| Guanine nucleotide-binding protein G(i) subunit alpha-2 | 6 | V-type proton ATPase subunit d 1 | 1 |
| Guanine nucleotide-binding protein G(I)/G(S)/G(T) subunit beta-1 | 3 | V-type proton ATPase subunit E 1 | 1 |
| Guanine nucleotide-binding protein-like 3 | 2 | Vesicle-associated membrane protein-associated protein B/C | 1 |
| Importin subunit alpha-1 | 2 | Voltage-dependent anion-selective channel protein 1 | 2 |
| Importin subunit alpha-3 | 2 | Voltage-dependent anion-selective channel protein 2 | 2 |
